# Central Nervous System Involvement in Adult-Onset Hemophagocytic Lymphohistiocytosis

**DOI:** 10.7759/cureus.14792

**Published:** 2021-05-01

**Authors:** Emmanuel Fohle, Felix Afriyie, Sammir S Dekowski

**Affiliations:** 1 Internal Medicine, University of North Dakota, Fargo, USA; 2 Internal Medicine, East Carolina University/Vidant Medical Center, Greenville, USA; 3 Internal Medicine, Newark Beth Israel Medical Center, Newark, USA

**Keywords:** hemophagocytic lymphohistiocytosis (hlh), altered mental state, fevers of unknown origin

## Abstract

Hemophagocytic lymphohistiocytosis (HLH) is a life-threatening disease marked by high cytokine levels, uncontrolled lymphocyte, and macrophage proliferation. It is generally a systemic disorder with varying degrees of central nervous system (CNS) involvement, with the vast majority of cases affecting children. We report a case of CNS-HLH in a 51-year-old male who initially presented with fevers, night sweats, fatigue, bilateral arthralgia, and altered mental status. Computed tomography (CT) of the chest, abdomen, and pelvis showed hepatosplenomegaly. Magnetic resonance imaging (MRI) of the brain showed enhancing lesions mainly in the right frontal lobe with a small hemorrhagic focus. An extensive workup for infectious, autoimmune, and neoplastic and genetic etiologies was only significant for cytopenia with markedly elevated C-reactive protein (CRP), ferritin, and lactate dehydrogenase (LDH), in addition to mild triglyceridemia. Bone marrow and liver biopsy revealed hemophagocytosis. Brain biopsy revealed no evidence of malignancy or infection. The patient was treated with high-dose dexamethasone and etoposide and fully recovered with resolution of all of HLH parameters and decrease/resolution of brain lesion. Clinicians should have a high index of suspicion for CNS-HLH in adults who present with sepsis-like illness with fevers, altered mental status, and cytopenia but negative cultures and unusual radiographic cerebral abnormalities so that early diagnosis and treatment can be initiated to prevent end-organ failure and death.

## Introduction

Hemophagocytic lymphohistiocytosis (HLH) is a clinical syndrome caused by a highly active but ineffective immune response. It includes impaired or absent function of natural killer (NK) cells and cytotoxic T cells, and the release of proinflammatory cytokines [1]. The primary form (familial HLH) is typically seen during infancy and early childhood, whereas adult-onset HLH is often secondary to an underlying disease such as infection, malignancy, or autoimmune disease [2]. Though HLH is typically a systemic disease with varying degrees of central nervous system (CNS) involvement, cases with primarily CNS involvement are more common in the pediatric population and manifest as meningitis, seizures, and optic neuritis [3]. We present a rare case of CNS-HLH in a middle-aged male with no genetic mutations suggestive of familial HLH and with no infectious, malignancy, or autoimmune disease.

## Case presentation

A 51-year-old male with a medical history of bipolar disorder type II presented to the clinic in June of 2019 with a three-month history of unintentional weight loss (25 pounds), bilateral knee pain, fatigue, exertional shortness of breath, and night sweats but no fevers. One month prior to this presentation, he was treated at a local walk-in clinic with doxycycline in a setting of a suspected tick bite while camping. In the clinic, he denied fevers or chills. Initial laboratory findings are summarized in Table 1. Since he had elevated inflammatory markers, he underwent autoimmune test and infectious disease workup including tickborne illness, flow cytometry on peripheral smear, and fungal serology, which were all negative. Fecal occult blood test was negative with subsequent normal colonoscopy examination. Computed tomography (CT) of the chest, abdomen, and pelvis showed mild splenomegaly but otherwise no acute pathology.

**Table 1:** Initial laboratory findings in summer 2019, December 2020, and summer 2020 WBC, white blood cell; RBC, red blood cell; MCV, mean corpuscular volume; BUN, blood urea nitrogen; ALT, alanine aminotransferase; AST, aspartate aminotransferase; CRP, C-reactive protein; LDH, lactate dehydrogenase

	Summer 2019	December 2019	Summer 2020	Reference range
WBC	5.9	6	5.8	3.6-10.3 x 10^3^/mcL
RBC	4.2	4.05	3.16	4.6-6.8 x 10^6^/mcL
Hemoglobin	12.4	12.2	9.3	13-15 g/dL
Hematocrit	38.1	35.7	29.5	40.0-50.0%
MCV	90.7	88.1	93.4	80.0-98.0 fL
Platelet	205	219	146	140-420 x 10^3^/mcL
Blood glucose	91	82	112	70-100 mg/dL
Sodium	136	135	132	135-145 mmol/L
Potassium	4.2	3.8	4.3	3.7-5.1 mmol/L
Chloride	104	106	102	96-110 mmol/L
Bicarbonate	28	21	24	22-32 mmol/L
BUN	12	14	10	6-24 mg/dL
Creatinine	1.13	1.05	0.97	0.6-1.3 mg/dL
Calcium	9.4	9.5	9.1	8.5-10.5 mg/dL
Bilirubin total	0.8	0.5	0.6	0.2-1.2 mg/dL
ALT	35	49	50	0-35 U/L
AST	27	33	64	0-35 U/L
CRP	80.3	20	85.1	0.0-8.0 mg/L
LDH	670	245	985	125-245 u/L
Ferritin	1,413	238	856	21-275 ng/mL
Total iron	31	45	35	65-175 ug/dL
Fibrinogen	444	400	410	200-450 mg/dL

In December of 2019, the patient was seen in the clinic as a routine follow-up during which he reported gradual symptoms resolution and stabilization of his weight over months without any specific treatment. He denied fevers, chills, or night sweats. He still had mild knee pain but was otherwise functional without any deficit. His labs at this time are shown in Table 1.

In June of 2020, he returned to the clinic with similar complaints of weight loss, fatigue, and bilateral knee pain with occasional fevers and night sweats. At this time, he had lost up to 15 pounds since the last visit. Repeat labs are shown in Table 1. There was a suspicion for HLH even though the lactate dehydrogenase (LDH) was not elevated enough to support this. The patient was referred to the hematology service.

**Figure 1:**
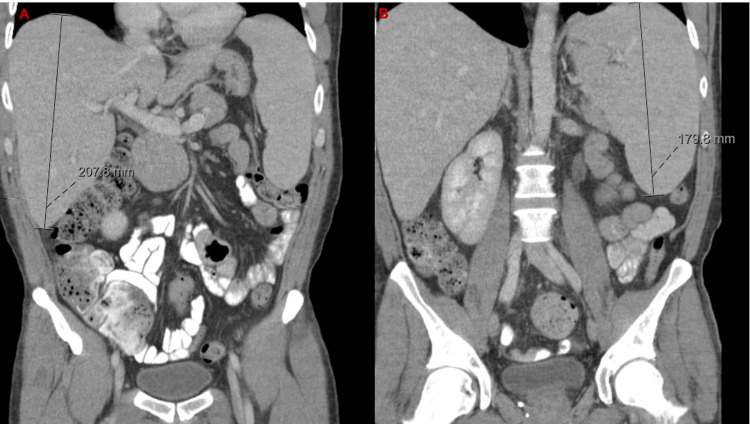
(A) Hepatomegaly. (B) Splenomegaly.

A week later, he woke up with severe shortness of breath, lightheadedness, and confusion, and was brought to the emergency department (ED). His vital signs included a heart rate of 100 bpm, blood pressure of 98/60 mmHg, temperature of 37.5°C (99.5°F), and pulse oximetry of 98% in room air. Physical examination was notable for an ill-appearing middle-aged male who was alert, oriented but occasionally confused. Respiratory examination revealed clear breath sounds bilaterally. Neurological examination showed no deficit. Venous blood gas was normal, D-dimer was 1.21 ug/mL FEU (normal < 0.49 ug/mL FEU), and haptoglobin was normal; the remainder of laboratory findings in the ED are summarized in Table 2. Electrocardiography (EKG) showed sinus tachycardia with a heart rate of 104 bpm. Chest X-ray was normal. Computed tomography angiography (CTA) of the chest did not show evidence of pulmonary embolism. CT of the abdomen and pelvis showed mild hepatosplenomegaly (Figures 1A, 1B). The patient was admitted to the hospital for further evaluation and management.

**Table 2:** Laboratory tests in the emergency department WBC, white blood cell; RBC, red blood cell; MCV, mean corpuscular volume; BUN, blood urea nitrogen; ALT, alanine aminotransferase; AST, aspartate aminotransferase; CRP, C-reactive protein; LDH, lactate dehydrogenase

	Emergency department visit	Reference range
WBC	3.5	3.6-10.3 x 10^3^/mcL
RBC	2.59	4.6-6.8 x 10^6^/mcL
Hemoglobin	7.5	13-15 g/dL
Hematocrit	23.8	40.0-50.0%
MCV	91.9	80.0-98.0 fL
Platelet	128	140-420 x 10^3^/mcL
Blood glucose	101	70-100 mg/dL
Sodium	129	135-145 mmol/L
Potassium	3.9	3.7-5.1 mmol/L
Chloride	101	96-110 mmol/L
Bicarbonate	24	22-32 mmol/L
BUN	13	6-24 mg/dL
Creatinine	1.33	0.6-1.3 mg/dL
Calcium	9	8.5-10.5 mg/dL
Bilirubin total	0.6	0.2-1.2 mg/dL
ALT	72	0-35 U/L
AST	71	0-35 U/L
CRP	103.1	0.0-8.0 mg/L
LDH	1,106	125-245 u/L
Ferritin	1,927	21-275 ng/mL
Total iron	18	65-175 ug/dL
Fibrinogen	430	200-450 mg/dL

Further infectious disease tests including human immunodeficiency virus (HIV), COVID-19, tuberculosis, Epstein-Barr virus (EBV), cytomegalovirus, syphilis, hepatitis panel, and West Nile Virus (WNV) were all negative. Serum protein electrophoresis (SPEP) was negative for monoclonal gammopathy. Differential diagnosis included slowly growing indolent splenic marginal zone non-Hodgkin lymphoma (NHL), occult myeloproliferative disease such as chronic myelomonocytic leukemia, and hairy cell leukemia, even though his previous flow cytometry was normal. Angioimmunoblastic T-cell lymphoma related NHL or gamma/delta hepatosplenic T-cell lymphoma were also considered though less likely given long clinical course of the patient’s symptoms. Low-grade HLH was also on the differential given the patient met 4/8 diagnostic criteria (hemophagocytosis, splenomegaly, high ferritin, and mildly increased triglyceride (156 mg/dL; normal 50-150 mg/dL).

**Figure 2:**
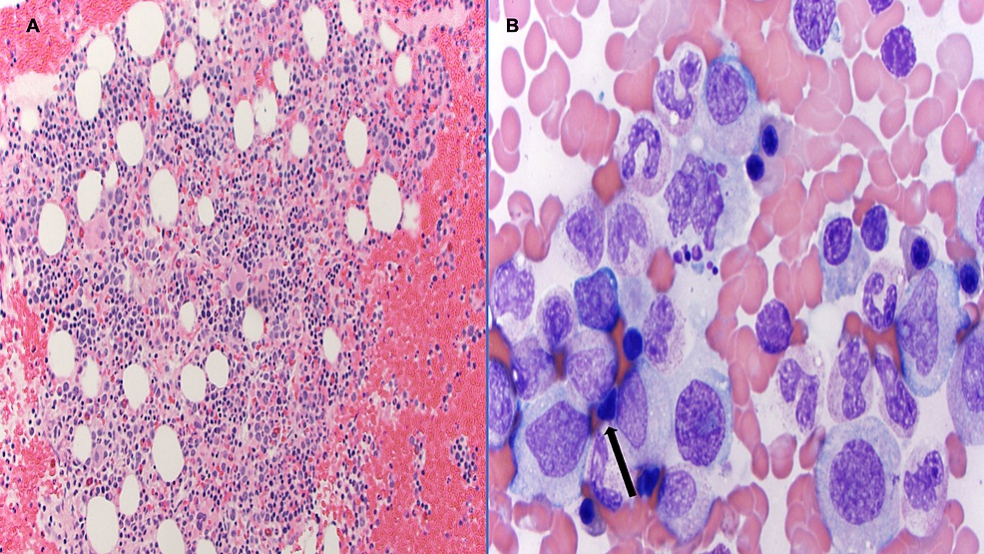
(A) Hypercellular marrow, bone marrow clot, x20. (B) Hemophagocytosis, bone marrow aspirate, x100 oil.

**Figure 3:**
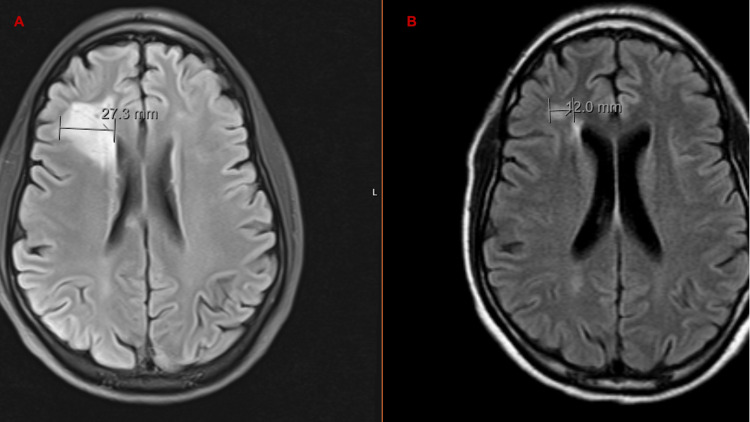
(A) MRI of the brain with enhancing lesion in the right frontal lobe. (B) MRI of the brain with resolving lesion.

The patient underwent bone marrow biopsy with aspiration, which revealed hypercellular marrow and evidence of hemophagocytosis, though no evidence of B-cell, T-cell lymphoma or Hodgkin lymphoma (Figures 2A, 2B) was found. The interleukin-2 receptor (IL-2)/soluble CD25 (sCD25) level was significantly elevated at 9,700 pg/mL (normal: 175-858.2 pg/mL). Magnetic resonance imaging (MRI) of the brain was obtained, which showed multiple abnormally enhancing lesions mainly in the right frontal lobe with a small hemorrhagic focus (Figure 3A). Cerebrospinal fluid (CSF) analysis was not suggestive of an infectious cause. A stereotactic needle brain biopsy revealed no evidence of malignancy or infection. Liver biopsy confirmed evidence of hemophagocytosis with no evidence of malignancy or lymphoma. A diagnosis of CNS- HLH was made, and the patient was started on high-dose dexamethasone 20 mg and etoposide 150 mg/m^2^ per the HLH-94 protocol for eight weeks alongside prophylactic BactrimÔ DS 800-160mg and acyclovir 400 mg. He had an excellent response with resolution of all his symptoms and normalizations of all HLH parameters (Table 3). Follow-up brain MRI showed decrease/resolution of previous parenchymal frontal abnormalities (Figure 3B). HLH genetic screening test showed no abnormalities.

**Table 3:** Laboratory findings after treatment WBC, white blood cell; RBC, red blood cell; MCV, mean corpuscular volume; BUN, blood urea nitrogen; ALT, alanine aminotransferase; AST, aspartate aminotransferase; CRP, C-reactive protein; LDH, lactate dehydrogenase

	Three months post-treatment	Six months post-treatment	Reference range
WBC	8.5	6.3	3.6-10.3 x 10^3^/mcL
RBC	3.42	5.26	4.6-6.8 x 10^6^/mcL
Hemoglobin	10.7	15.9	13-15 g/dL
Hematocrit	32.9	46.5	40.0-50.0%
MCV	96.2	88.4	80.0-98.0 fL
Platelet	229	229	140-420 x 10^3^/mcL
Blood glucose	91	82	70-100 mg/dL
Sodium	94	84	135-145 mmol/L
Potassium	3.7	4.2	3.7-5.1 mmol/L
Chloride	106	108	96-110 mmol/L
Bicarbonate	28	30	22-32 mmol/L
BUN	20	24	6-24 mg/dL
Creatinine	1.18	1.13	0.6-1.3 mg/dL
Calcium	9.1	9.1	8.5-10.5 mg/dL
Bilirubin total	0.4	0.5	0.2-1.2 mg/dL
ALT	15	40	0-35 U/L
AST	14	19	0-35 U/L
CRP	3.7	0.8	0.0-8.0 mg/L
LDH	233	165	125-245 u/L
Ferritin	299	70	21-275 ng/mL
Total iron	33	40	65-175 ug/dL
Fibrinogen	420	440	200-450 mg/dL

## Discussion

HLH is a hyper-inflammatory condition induced by the overactivation of lymphocytes and macrophages, as well as elevated cytokine levels [3,4]. There are two forms of HLH: a primary form that manifests in children with established genetic abnormalities affecting the cytotoxic activity of NK cells and T cells, and a secondary form that manifests in adults with an underlying disorder, such as infection or malignancy, but no known genetic abnormality [3,5,6]. Fever, malaise, hepatosplenomegaly, jaundice, lymphadenopathy, cytopenia, and a number of neurological symptoms are some of the clinical manifestations [4,7]. Changes in mental state, seizures, meningitis, ataxia, and cranial nerve palsies are all signs of CNS involvement [3,8,9]. The majority of such cases occur in the pediatric population up to 75% and represent primary CNS-HLH [3,4].

The frequency of CNS-HLH involvement in adults has been studied only in a few cases. CNS-HLH was reported in 73% of patients with HLH at the time of diagnosis in one case series of 34 patients, and CNS complications eventually developed in 100% of HLH patients who did not receive bone marrow transplantation [10]. In a separate study, 56% of 30 patients had CNS involvement, with 46% of those presenting with neurological symptoms [11]. In another study, 30% of CNS-HLH patients had an underlying autoimmune disorder [12]. In the HLH-94 study, 37% of the 193 patients had neurological symptoms at the time of diagnosis and 52% had abnormal CSF [13]. Another major retrospective study discovered that 10% of 289 adult HLH patients had CNS involvement and that CSF and neuroimaging results are usually unspecific [9].

HLH is diagnosed clinically by either having a proven genetic mutation known to be associated with HLH or by meeting 5/8 clinical criteria of fever (≥38.50 C), splenomegaly, cytopenia of at least two cell lines (hemoglobin < 9 g/dL [for infants <4 weeks, hemoglobin < 10 g/dL], platelets < 100,000/microL, absolute neutrophil count < 1000/microL), hypertriglyceridemia (>265 mg/dL) and/or hypofibrinogenemia (<150 mg/dL), hyperferritinemia (>500 ng/mL), high levels of soluble IL-2 receptor, low or absent NK cell activity, and pathological evidence of hemophagocytosis in the bone marrow, spleen, lymph, node or liver [4,12].

In our case, the patient met 6/8 criteria (hemophagocytosis, splenomegaly, high ferritin, slightly increased triglyceride, elevated IL-2/soluble CD25, and hemophagocytosis on liver biopsy) and had an enhancing lesion on brain MRI, indicating CNS-HLH. Dexamethasone, etoposide, and cyclosporine A, as well as intrathecal methotrexate, are used in the HLH-94 protocol [4]. In genetic or refractory case, a stem cell transplant may be considered [14]. Since he tested negative for genetic changes, he most likely had a case of acquired HLH with unknown causes such as infection, malignancy, autoimmune disease, or medications. Prior to his initial presentation in the summer of 2019, he was briefly treated for suspected tick bite with doxycycline, but testing for tick-borne diseases was negative. It is possible that he was exposed to an environmental trigger such as a virus for a short period of time, which might have precipitated CNS-HLH. He was successfully treated with high-dose dexamethasone and etoposide and fully recovered with resolution of all HLH parameters and improvement in previous parenchymal frontal abnormalities on MRI.

## Conclusions

In conclusion, astute clinicians should have a high index of suspicion for HLH in adults who present with unexplained fevers, altered mental status, and cytopenia so that adequate testing can be performed rapidly and early treatment can be initiated to control hypercytokinemia, which can result in end-organ failure and death if left untreated. Imaging results should also be associated with clinical symptoms in addition to laboratory and pathological findings.
